# Automating hock wound detection in dairy cattle

**DOI:** 10.3168/jdsc.2024-0671

**Published:** 2024-11-05

**Authors:** W. Flanders, P.S. Basran, M. Wieland

**Affiliations:** 1 Department of Clinical Sciences, College of Veterinary Medicine, Cornell University, Ithaca, NY 14853; 2 Department of Population Medicine and Diagnostic Sciences, College of Veterinary Medicine, Cornell University, Ithaca, NY 14853

## Abstract

Hock scoring in dairy cattle is a crucial welfare assessment tool used to evaluate the condition of a cow's hocks, particularly for signs of injury, swelling, or lesions. These scores provide insight into the overall well-being of the animals and are essential for ensuring proper management and housing conditions. Accurate hock scoring is vital because it can indicate issues such as poor bedding quality or inadequate space, which directly affect the health and productivity of the herd. Traditionally, hock scoring is performed manually by trained observers. However, consistency in scoring can be a challenge. Two studies were conducted to quantify inconsistency in hock scoring. In one study, manual scoring reproducibility was measured. In the second study, manual and video scoring repeatability was measured. Repeatability was quantified with a weighted Cohen's kappa metric. Manual scoring was found to be inconsistent but more consistent than video scoring. This variability highlights the need for a more reliable, objective method of scoring. To address this, we explored the automation of hock score detection using artificial intelligence. Specifically, we employed a simple U-net semantic segmentation algorithm to detect wounds on the hocks without classifying them into specific categories. Automating the detection process can reduce observer bias, improve consistency, and allow for continuous monitoring of large herds. This approach holds promise for enhancing animal welfare by providing a more efficient and accurate method of assessing hock health in dairy cattle.

## Highlights

• Hock scoring is performed to identify injuries, swelling, and lesions.
• Hock scoring is a critical tool for measuring dairy cattle welfare.
• Manual scoring shows significant variability between in-person observers.
• Manual scoring also shows significant variability between in-person and video assessments.

## Graphical Abstract

**Figure:**
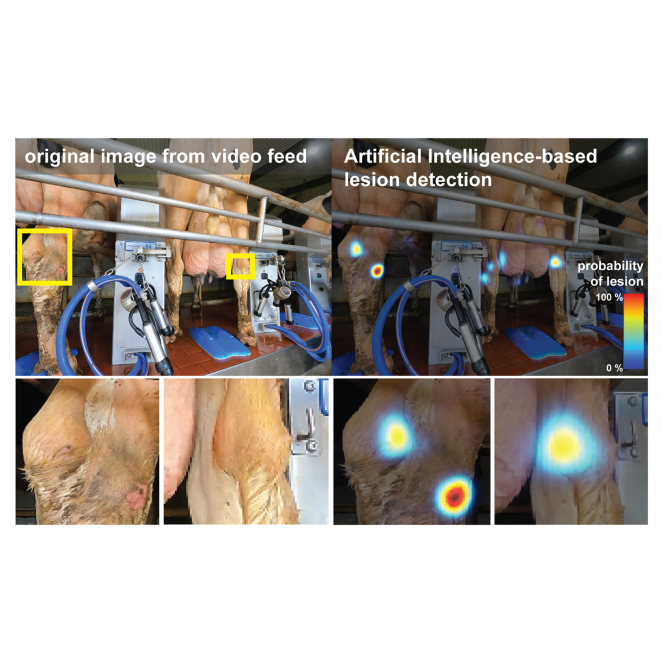
**Summary:** Artificial intelligence-based detection was implemented using a U-net semantic segmentation algorithm to identify wounds on cattle hocks, and could distinguish lesions with an area under the curve of 95%.

Hock scoring is a welfare assessment tool used in dairy farming to evaluate the condition of a cow's hocks, which are the joints at the rear legs, specifically the tuber calcis and tarsal joint. The prevalence of hock lesions, often exceeding 50%, is strongly associated with prolonged exposure to abrasive surfaces, high local pressure, and poor hygiene, all of which can lead to health issues, including lameness and subsequent economic losses (Kester et al., 2014). Lameness, which is closely linked to hock condition, affects dairy cow welfare and productivity, highlighting the importance of accurate and consistent hock scoring as an animal-based measure in welfare assessments (Sadiq et al., 2017). Moreover, the type of bedding used in stalls can influence the odds of developing hock lesions, with studies showing that bedding materials like recycled manure solids can lead to cleaner hocks and potentially reduce the incidence of lesions, although the relationship is complex and multifactorial (Fréchette et al., 2022). Thus, hock scoring remains a vital tool in managing and improving the welfare of dairy cattle, necessitating reliable methods for its accurate assessment.

Hock scoring is done in 3 categories: The scoring system typically assigns a score of 1 for hocks with no visible damage, indicating healthy, unblemished skin. A score of 2 is given when abrasions are present, which suggests mild skin damage, often from friction or pressure against hard surfaces. A score of 3 is assigned to hocks with lesions indicating more severe injury, such as swelling or open wounds.

Hock scoring is performed manually by a trained observer. This process is time-consuming and is subject to inconsistency. Computer-vision based detection has been used to assess items like body condition score and teat condition (Porter et al., 2021). We hypothesized that hock scoring can be performed reliably using machine learning.

Our study was conducted on June 20, 2024, at a commercial dairy farm located near Ithaca, New York, where they milk 4,900 cows and raise 3,900 head of youngstock. The farm belongs to the client base of Quality Milk Production Services and was selected based on its willingness to participate in the study. Holstein cows were housed in freestall pens, bedded with manure solids, fed a TMR, and milked 3 times daily in a 100-stall rotary parlor. All procedures involving animals were reviewed and approved by the Institutional Animal Care and Use Committee of Cornell University (IACUC protocol number: 2020–0004). During a milking session, 2 observers, an expert and a trainee, independently manually scored 1,000 cows, assigning scores of 1, 2, or 3 to each cow depending on their hock condition. A score of 1 indicates no lesion, a score of 2 indicates a lesion, and a score of 3 indicates an open wound or swelling. Additionally, the same 1,000 cows were video recorded. In the rotary dairy parlor setup, a GoPro camera (GoPro Inc., San Mateo, CA) was strategically positioned to capture detailed footage of the cows after the milking unit had detached from the udder. The camera was set up to focus on the cows as they pass by after the milking unit has disengaged, providing multiple angles of the cows' backsides. This placement ensures a thorough view of the hocks from different perspectives as the cows move through the parlor, allowing for a comprehensive assessment of their condition. At a later time, one of the observers repeated the hock score assessment using the video recording of the milking session.

The interoperator reproducibility between the 2 observers and between the video and in-person scoring was quantified with a weighted Cohen's kappa. Cohen's kappa is a statistical measure used to assess the level of agreement between 2 observers beyond what might be expected by chance alone (McHugh 2012). Calculations were performed in Matlab (version 2023a, MathWorks).

Once the interoperator variability was assessed in the 2 scenarios, in person, and with a video, the scores were used to produce a dataset for training a machine learning model. The trainee manually created binary masks of the exact location and contour of each hock lesion that the expert identified in the video. A semantic segmentation approach was chosen, focusing on automatically detecting and highlighting the existence and location of any lesion, wound, or injury on the hocks, regardless of its severity. Semantic segmentation is the process of highlighting the position and outline of a feature of interest (Ronneberger, 2017). We chose the U-net for semantic segmentation of hock lesions due to its effectiveness in handling complex image segmentation tasks and its ability to preserve spatial information through its encoder-decoder structure. Its design, with skip connections, allows for precise delineation of lesions, making it well-suited for identifying and segmenting hock lesions from high-resolution footage. The U-net detection algorithm was implemented using WebGL and JavaScript, utilizing GPU shaders (WebGL V2, Khronos Group; JavaScript ES7, Oracle)—programs optimized for parallel computation on graphics hardware—to perform convolutional, deconvolutional, max-pooling, and weight-matrix updates efficiently. This approach avoided additional libraries or interfaces, ensuring a highly customized and efficient processing pipeline. The U-net architecture, as detailed by Ronneberger (2017), is a specialized convolutional neural network designed for semantic segmentation tasks in biomedical imaging. It features a symmetric encoder-decoder structure with skip connections that facilitate the preservation and utilization of spatial context, making it particularly effective for tasks requiring precise delineation of structures. U-net is widely used in applications such as tumor boundary detection in magnetic resonance imaging (MRI) scans, cell segmentation in microscopy images, and organ segmentation in computed tomography (CT) scans, where accurate identification and segmentation of specific anatomical structures are essential.

The model was trained with a dataset consisting of 650 manually labeled lesions. These lesions were annotated by meticulously creating wound masks, which were crucial for training the network to recognize and segment lesions accurately. The real-time processing of high-resolution video footage was achieved using a 256 × 256 pixel sliding window, allowing for continuous analysis and generating heat maps that display the probability and severity of detected lesions.

The architecture of the U-net was carefully constructed to include an input layer of dimensions 256 × 256 × 3, followed by a sequence of pooling, activation, and max-pooling layers. These layers progressively reduce the spatial dimensions while increasing the depth of feature maps. The smallest and deepest layer is 8 × 8 × 64. The network then employs deconvolutional layers to upsample the feature maps, restoring the spatial dimensions and refining the segmentation outputs. This architecture enables effective segmentation and accurate lesion detection, addressing the complexities of real-time video analysis and enhancing the model's ability to deliver actionable insights for wound assessment.

The final output of the wound segmentation is passed through a Gaussian blur, and a rainbow heat map is applied to the blended intensity map. The heat map is superimposed onto the video feed, highlighting the location and perceived severity of the wound.

In the first study, which examined interobserver reproducibility during in-person assessments, the weighted Cohen's kappa with standard deviation was 0.558 ± 0.040. Although this value indicates a relatively high level of agreement between the 2 raters, it also suggests that there is still some level of inconsistency in their evaluations (Table 1). Although the agreement is above the threshold of chance, the presence of discrepancies points to potential variability in the scoring system.

**Table 1:** Cross-correlation table of expert in person versus trainee in person scoring of hock lesions^1^

Expert	Trainee		
	1	2	3
1	1,161	196	91
2	3	94	72
3	0	3	10

1 Score 1 is no wound. Score 2 is a lesion. Score 3 is an open wound.

In the second study, the repeatability of evaluations conducted by the same observer, both in person and via video, was assessed (Table 2). The weighted Cohen's kappa coefficient in this scenario was 0.453 ± 0.058, reflecting a moderate level of agreement. This result highlights a notable drop in consistency when transitioning from in-person assessments to video-based evaluations. The reduction in agreement underscores important challenges associated with the video modality, potentially due to factors such as reduced visual clarity or limited contextual information.

**Table 2:** Cross-correlation table of expert in person versus expert through video scoring of hock lesions^1^

Expert in person	Expert through video		
	1	2	3
1	476	118	50
2	109	131	30
3	30	19	23

1 Score 1 is no wound. Score 2 is a lesion. Score 3 is an open wound.

The U-net was successful in identifying the location of wounds. The receiver operating characteristic (**ROC**) curve is a graphical representation that illustrates the trade-off between the true positive rate and the false positive rate across different threshold settings for a classification model. The area under the ROC curve (**AUC**) quantifies the model's overall performance (Figure 1). An AUC of 0.95, as observed in our results, signifies an exceptional model performance. This high AUC value indicates that the model is highly effective at distinguishing between positive and negative cases, with only a 5% probability of misclassifying a randomly chosen positive instance as negative and vice versa. Such a result demonstrates strong discriminatory power and reliability in classification tasks, reflecting a well-performing model. The AUC calculations were performed with Python.

**Figure 1:**
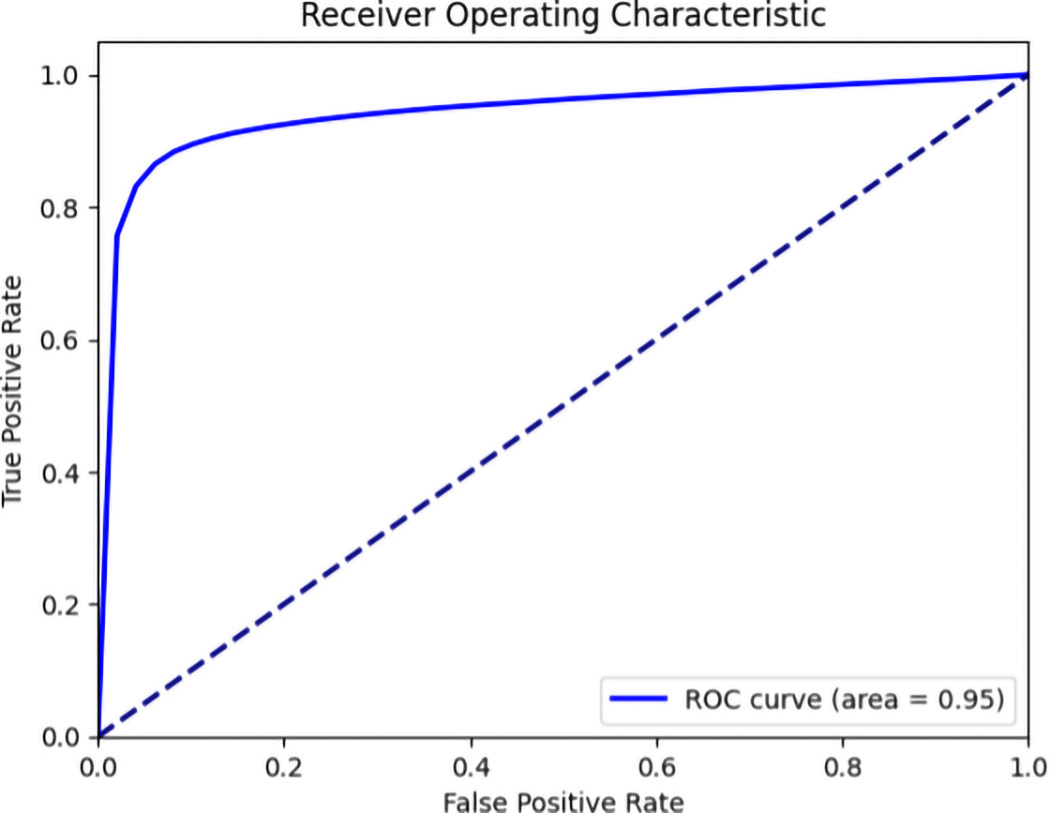
Receiver operating characteristic (ROC) curve for lesion detection (equal to or above a score of 2) using the U-Net semantic segmentation algorithm (solid), along with the line of identify (dash), which represents a random guess. The 95% CI are too small to plot on the ROC curve.

While hock lesions are typically scored with scores of 2 and 3, the U-net outputs a probability of a wound being present. When the wound is evident in the network, the heat map shows a more intense color. The U-net's wound severity metric does not necessarily correspond to the 2 and 3 classification metrics used clinically but could still be a valuable metric for assessing hock health.

Hock scoring is crucial for monitoring and managing the welfare of dairy cattle, as it provides valuable insights into the prevalence and severity of lesions that can affect animal health and productivity. Manual scoring is potentially unreliable. Automating this process is essential for increasing efficiency and consistency, reducing the time and labor required for manual assessments. By employing a U-net for detecting wounds from video footage, this study demonstrated the effectiveness of advanced semantic segmentation techniques in automating hock scoring. The U-net's ability to accurately identify and segment lesions from high-resolution video data underscores its potential to enhance the reliability and scalability of cattle welfare assessments, ultimately contributing to improved animal care and management practices.
